# Illinois

**Published:** 1897-03

**Authors:** 


*Illinois.* A bill intended to regulate the practice of veterinary medicine and surgery makes it unlawful for any person to practise without having previously obtained a diploma from a college duly authorized to graduate students in veterinary medicine and surgery, or the person shall have been in continuous practice exclusively as a veterinary practitioner in that State for a period of ten years; those practising less than ten years must pass an examination before the State Veterinary Board. Said board to consist of five duly qualified practitioners, to be appointed by the Governor, with the advice and consent of the Senate, and to hold office for five years. Not more than two graduates of any one college, and no professor or instructor of any college, shall serve on this board. The board shall meet every six months in the cities of Chicago and Springfield, alternately; each member to receive five dollars per day and all necessary expenses while engaged in the duties of office; the same to be paid from fees received by the secretary. Said board shall examine all diplomas as to their genuineness, and an affidavit shall be made by the holder that he is the person mentioned therein; said affidavit being sufficient guarantee that it is genuine. The board shall examine non-graduates by written or oral examination, or both. An examination fee of five dollars is to be paid by the non-graduate presenting himself, the same to be returned in case of failure. The board shall grant license to practise, for which they shall receive a fee of five dollars, having the power to refuse the license to any applicant on the ground of his having been guilty of gross immorality or gross malpractice; but no license shall be revoked without citing the party charged before said board by notice in writing, and upon evidence taken by said board in his presence, and after he has had opportunity to disprove such charges. Said license must be recorded in the office of the clerk of the county, for which the holder shall pay the usual fee for making said record. For any failure, neglect, or refusal to register as above for a period of six months he shall forfeit his license, which cannot be restored except on the payment to said board of the sum of twenty-five dollars penalty. Any person is regarded as practising according to the meaning of this Act who shall publicly profess to be a veterinary surgeon or appends to his name any initials or title implying qualifications to practise, or who prescribes for or treats sick or injured domestic animals. Nothing in this Act prohibits veterinary students from prescribing under the supervision of a preceptor nor to prohibit gratuitous services. The fine for practising without a license shall not be less than twenty-five dollars nor more than one hundred dollars. Any person filing or attempting to file a diploma or a certificate of another shall be subject to such fine and imprisonment as is made and provided by the statutes of the State for the crime of forgery. One-half of the fines provided for shall be paid into the county treasury and the other half to the prosecutor.

